# Using QSM to investigate the iron deposition patterns in deep gray matter nuclei during the aging process in healthy populations

**DOI:** 10.3389/fnagi.2026.1839435

**Published:** 2026-08-18

**Authors:** Xingyu Wang, Yiming Wang, Lin Liu, Ziqi Wang, Qingfeng Zhu, Duo Gao, Xuefang Han, Huandi Lv, Song Zhao, Hong Song, Xueshan Cao, Zuojun Geng

**Affiliations:** 1 Department of Medical Imaging, The Second Hospital of Hebei Medical University, Shijiazhuang, China; 2 Department of Radiology, The Affiliated Hospital, Southwest Medical University, Luzhou, Sichuan, China; 3 Department of Occupational Health and Environmental Health, School of Public Health, Hebei Medical University, Shijiazhuang, China; 4 Hebei Key Laboratory of Environment and Human Health, Shijiazhuang, China; 5 School of Medical Imaging, Hebei Medical University, Shijiazhuang, China; 6 Beijing Institute of Technology, Beijing, China

**Keywords:** brain iron deposition, deep gray matter nuclei, magnetic resonance imaging, magnetic susceptibility value, quantitative susceptibility mapping

## Abstract

**Objective:**

We utilized 3.0T quantitative susceptibility mapping (QSM) technology to simultaneously investigate (a) the relationship between susceptibility values of deep gray matter (DGM) nuclei and age in healthy populations throughout the aging process, and (b) the correlation of iron deposition among DGM nuclei and its potential clinical significance.

**Methods:**

QSM images of DGM nuclei were obtained from 100 healthy participants using a 3.0T MRI scanner. Susceptibility values of corresponding DGM nuclei were acquired by manually delineating regions of interest (ROIs). Pearson correlation analysis and univariate regression analysis were performed between age and the susceptibility values of each DGM nucleus. Partial correlation analysis was employed to investigate the relationships between mean susceptibility values across multiple nuclei.

**Results:**

Except for the CN, the susceptibility values of the other five DGM nuclei showed a significant positive correlation with age. Among them, the PUT had the strongest correlation with age (*r* = 0.71, *p* < 0.001), and the iron deposition rate in the PUT increased the most with age (linear regression slope = 0.93 ppb/year). Additionally, the average susceptibility values between multiple pairs of DGM nuclei, including SN and GP (*r* = 0.59, *p* < 0.001), SN and DN (*r* = 0.46, *p* < 0.001), etc., still shows a significant positive correlation after controlling for age.

**Conclusion:**

Iron deposition in the PUT exhibits the most significant age dependence; the correlation of iron deposition among nuclei may indicate that specific nuclei share the same iron deposition patterns or are involved in the same pathological mechanisms.

## Introduction

Iron is the most abundant transition metal in the brain, existing in the human body in the forms of heme iron and non-heme iron. The former is primarily associated with blood circulation or accumulation, while the latter is found in nearly all cells and plays a crucial role in essential biological processes in various brain cells, including oxygen transport, DNA synthesis and repair, mitochondrial respiration, myelin synthesis, and neurotransmitter synthesis and metabolism (Hentze et al., 2004). Iron homeostasis is essential for maintaining normal physiological brain functions. When brain iron levels exceed the cellular iron-chelating capacity of storage proteins or other molecules, leading to iron homeostasis disorders, toxic free iron can react with oxygen to generate neurotoxic free radicals, which may promote membrane lipid peroxidation and the accumulation of lipofuscin in neurons (Koeppen, 2003), leading to neuronal cell death and axonal degeneration (Kurz et al., 2008), thereby accelerating the progression of neurodegenerative diseases (Cheng et al., 2022).

The distribution of brain iron in healthy adults is uneven. Early postmortem anatomical studies of brain tissue have reported that iron deposition is particularly pronounced in the deep gray matter (DGM) regions associated with motor control, specifically the globus pallidus (GP), caudate nucleus (CN), putamen (PUT), and substantia nigra (SN). Furthermore, iron content exhibits a continuous increase from childhood to adolescence. (Spatz, 1922). DGM nuclei serve as the core of many neural circuits connecting multiple locations in the cerebral cortex (Pagnozzi et al., 2019),playing a crucial role in processing vital physiological processes such as motor initiation, cognition, and memory. Excessive iron deposition in DGM nuclei is associated with neurodegenerative diseases, such as Parkinson’s disease (PD), atypical parkinsonian disorders (APDs), Alzheimer’s disease (AD), etc. It remains unclear whether the iron deposition observed in neurodegenerative diseases is a primary event or a secondary effect (Zucca et al., 2017). The process of aging significantly contributes to the risk of neurodegeneration, with the age-associated accumulation of iron potentially playing a crucial role in the mechanisms underlying these diseases. Therefore, a reliable and quantitative *in vivo* non-invasive assessment of iron deposition in DGM nuclei during normal aging to determine normal iron content can provide a reference for neurodegenerative disease research and help further clarify the role of iron in neurodegenerative diseases.

In contrast to other MRI methods sensitive to iron, including R2, R2*, field-dependent relaxation imaging (FDRI), and susceptibility-weighted imaging (SWI), quantitative susceptibility mapping (QSM) is, in principle, unaffected by water content, echo time, or field strength, thus overcoming the shortcomings of these technologies. QSM enables the true quantification of iron deposition (Marxreiter et al., 2023), which is particularly useful for studying iron-rich DGM nuclei.

Currently, numerous studies utilizing QSM have reported age-related increases in iron deposition within the DGM nuclei. However, the correlation between the susceptibility values of specific nuclei and age remains inconsistent. The GP is particularly controversial, as some studies have identified a significant positive correlation between GP susceptibility values and age (Taege et al., 2019; Zhou et al., 2020), while Acosta-Cabroner et al. and Li et al. did not observe such a correlation (Acosta-Cabronero et al., 2016; Li et al., 2015). Additionally, previous research has predominantly concentrated on DGM nuclei within the striatum and midbrain, yielding few and inconsistent findings regarding the correlation between susceptibility values of the cerebellar dentate nucleus (DN) and age (Madden and Merenstein, 2023).

Importantly, although age-related patterns of iron deposition in DGM nuclei have been extensively studied, inter-nuclear correlations in iron deposition remain underexplored. Specifically, it remains unclear whether iron deposition across different DGM nuclei exhibits coordinated characteristics, and whether such coordinated variations reflect underlying neural circuit interactions. Addressing this research gap may provide new clues and insights into the potential patterns of brain iron dyshomeostasis and its intrinsic association with neurodegenerative diseases

Therefore, this study aims to utilize QSM imaging to investigate the correlation between susceptibility values of DGM nuclei and age in healthy populations, while also constructing corresponding age-dependent trajectories. Meanwhile, this research seeks to assess whether there is a correlation in iron deposition among the DGM nuclei.

## Materials and methods

### Participants

From March 2024 to April 2025, a total of 115 healthy adults were recruited, all of whom signed the corresponding informed consent forms prior to enrollment. The study received approval from the Ethics Committee of the Second Hospital of Hebei Medical University. Participants were excluded if they had contraindications for MRI (e.g., pacemakers, aneurysm clips, inner ear implants, etc.), claustrophobia, a history of mental disorders, head trauma, or any conditions affecting brain iron content (e.g., PD, AD, restless legs syndrome, hepatic encephalopathy, etc.). Two experienced neuroradiologists evaluated the MRI scans, and participants were excluded if their images exhibited severe artifacts, obvious intracranial positive lesions, or multiple focal abnormal signals.

### MRI acquisition

A GE Signa Architect 3.0T superconducting MRI scanner equipped with a 48-channel head coil was utilized to conduct conventional MR sequences as well as QSM functional sequence scans. The scanning baseline was defined as the horizontal line connecting the anterior and posterior commissures, with the scanning range extending from the foramen magnum to the vertex of the skull. Volunteers were positioned in a supine posture. Prior to the examination, cotton balls were inserted into both ear canals to provide hearing protection, and foam pads were strategically placed on either side of each volunteer’s head to minimize head movement. The scanning parameters and sequences were as follows: 3D-T1WI parameters: Repetition time (TR) = 6.2 ms, echo time (TE) = 2.4 ms, Field of view (FOV) = 256 mm × 256 mm, number of slices = 160, slice thickness = 1 mm, flip angle (FA) = 8°. QSM parameters: TR = 27.2 ms, TE = 1.9 ms, FOV = 256 mm × 256 mm, number of slices = 130, slice thickness = 1 mm, FA = 12°. MAGIC parameters: TR = 4,150 ms, TE = 18.6 ms, FOV = 240 mm × 240 mm, number of slices = 30, slice thickness = 4 mm, Slice spacing = 1 mm. Scanning sequence: Since the MAGIC sequence generates synthetic images after scanning, which can automatically reconstruct T1FLAIR, T2, T2FLIR, T2STIR, and PDWI images, the MAGIC sequence was acquired first. Subjects were screened according to inclusion and exclusion criteria, and those meeting the inclusion criteria proceeded to the 3D-T1WI and QSM sequence scans.

### Data processing

**FIGURE 1:**
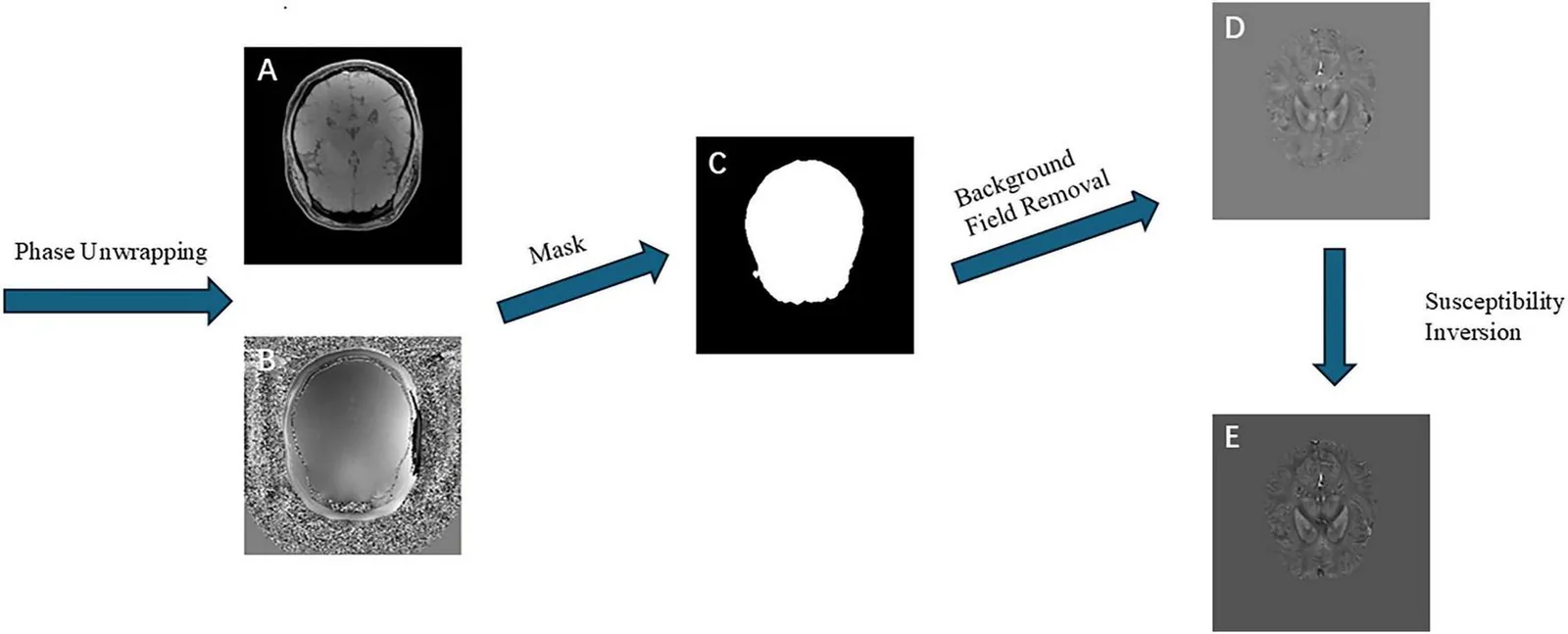
The post-processing flowchart of QSM. **(A)** Original Magnitude Image; **(B)** original phase image; **(C)** mask generated for skull removal; **(D)** local tissue field map obtained after removing background fields (such as external interferences at air-tissue interfaces) Produced by Magnetic Substances within the Brain; **(E)** final QSM image obtained through susceptibility inversion.

The scanned QSM images were imported into MATLAB (R2016b) software, utilizing the STI Suite v3.0 plugin^1^ for image post-processing. The reconstruction steps encompassed phase unwrapping, brain mask generation, background field removal, and susceptibility inversion. Briefly, a Laplacian-based algorithm was employed for phase unwrapping. Background field removal was subsequently performed using the 3D V-SHARP algorithm with a smoothing kernel size (smvsize) of 12. Finally, dipole inversion was conducted via the STAR-QSM algorithm with default parameters to generate the final susceptibility maps, which were stored in Nii format. The QSM post-processing procedure is illustrated in Figure 1.

**FIGURE 2:**
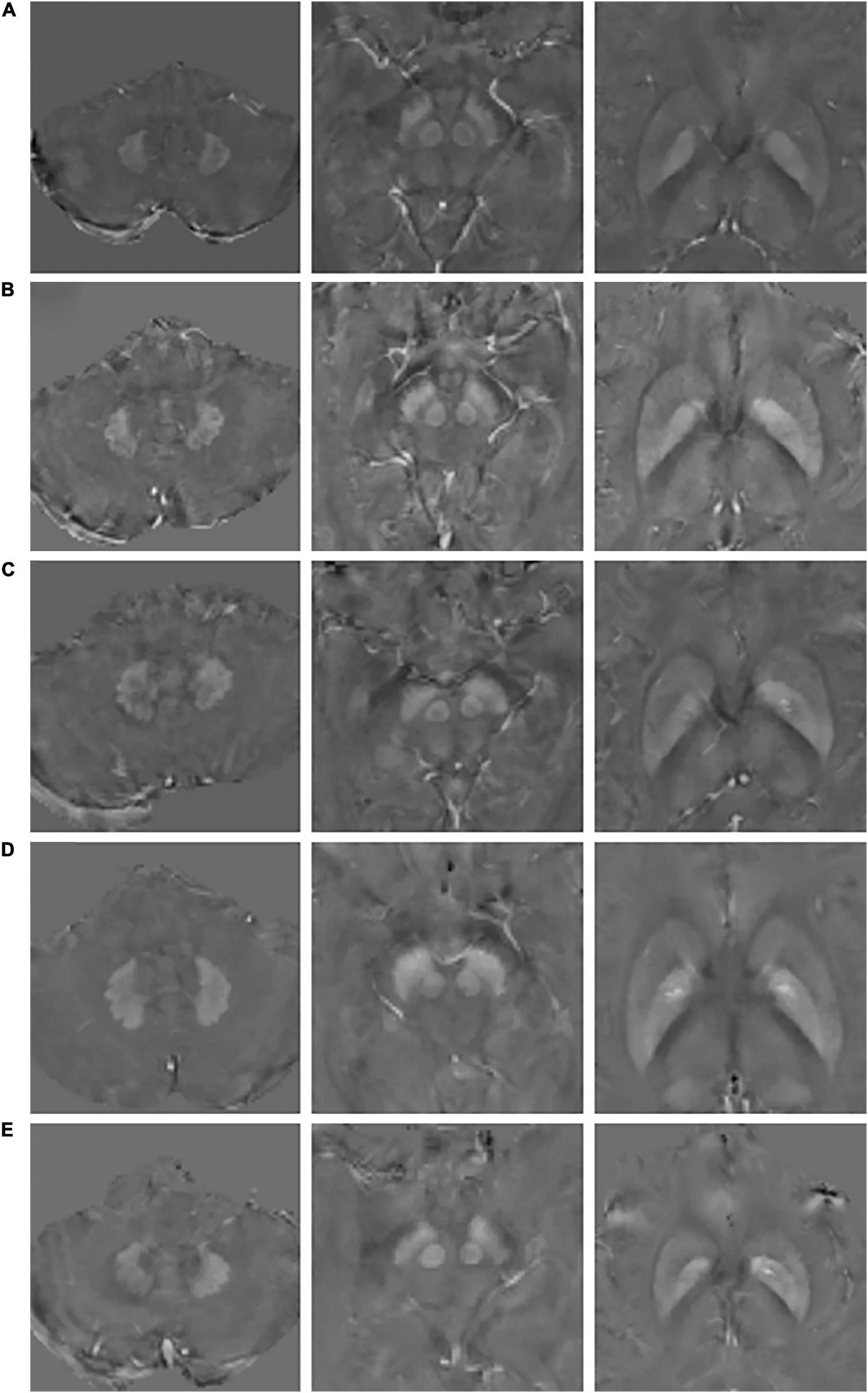
QSM images of five male volunteers of different ages. **(A)** 23-year-old male; **(B)** 39-year-old male; **(C)** 43-year-old male; **(D)** 52-year-old male; **(E)** 66-year-old male.

**FIGURE 3:**
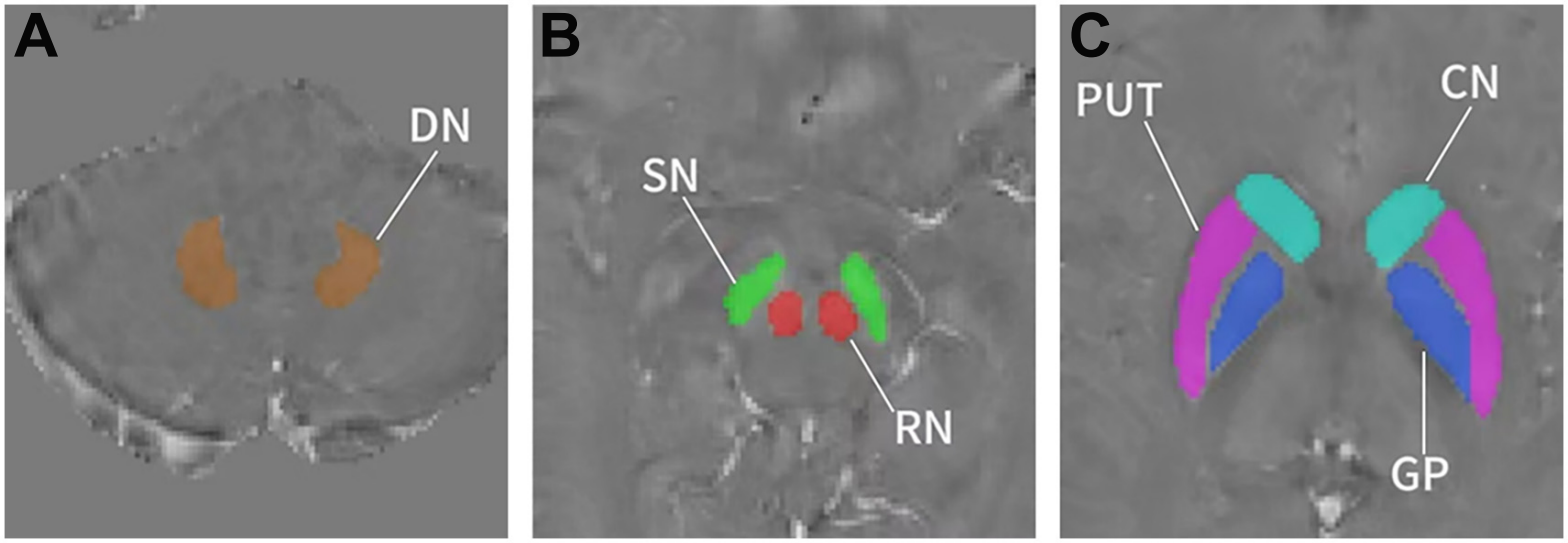
Schematic diagram of manually segmented ROIs delineated on QSM images. **(a)** The brown area represents the dentate nuclei (DN). **(b)** The red area represents the red nucleus (RN), and the green area represents the substantia nigra (SN). **(c)** The cyan area represents the head of the caudate nucleus (CN), the blue area represents the globus pallidus (GP), and the purple area represents the putamen (PUT).

The processed QSM images (Figure 2) were imported into ITK-SNAP (version 4.0.1) software (Yushkevich et al., 2006). Two experienced radiologists manually delineated the ROIs on the axial images, specifically the SN, red nucleus (RN), DN, GP, PUT, and CN (Figure 3). During the delineation process, the axial slice exhibiting the largest and clearest representation of each nucleus was selected as the ROI. The complete contour of each ROI was manually drawn to obtain the susceptibility values corresponding to the respective nuclei. Three consecutive slices were contoured, and the bilateral mean value was calculated to serve as the final statistical value. Care was taken to avoid air-cranium interfaces, blood vessels, and calcifications during the delineation process. The QSM data were reported in parts per billion (ppb).

### Statistical analysis

Statistical analyses were performed using IBM SPSS Statistics version 29.0.1.0. The inter-observer segmentation variability between the two researchers was evaluated using the intraclass correlation coefficient (ICC). An ICC value exceeding 0.9 indicates excellent consistency, while values ranging from 0.75 to 0.9 signify good consistency. To explore the relationship between the mean susceptibility values of each DGM nucleus and age, Pearson correlation analysis was utilized. Furthermore, partial correlation analysis was conducted to examine the relationships of iron deposition among multiple DGM nuclei. The strength of the Pearson correlation coefficient (r) was classified based on its absolute value according to the following criteria: 0.00–0.19 as “very weak,” 0.20–0.39 as “weak,” 0.40–0.59 as “moderate,” 0.60–0.79 as “strong,” and 0.80–1.0 as “very strong.” An independent samples *t*-test was employed to assess whether a significant difference existed in age between male and female experimental participants. Univariate regression analyses were performed between the mean susceptibility values of each DGM nucleus and age using linear and quadratic models. The best-fitting model was determined by comparing both the R^2^ values and the Akaike information criterion (AIC) across models Since multiple comparisons were performed, we applied the Bonferroni method for correction to avoid type-I error.

## Results

### Subject characteristics

**FIGURE 4:**
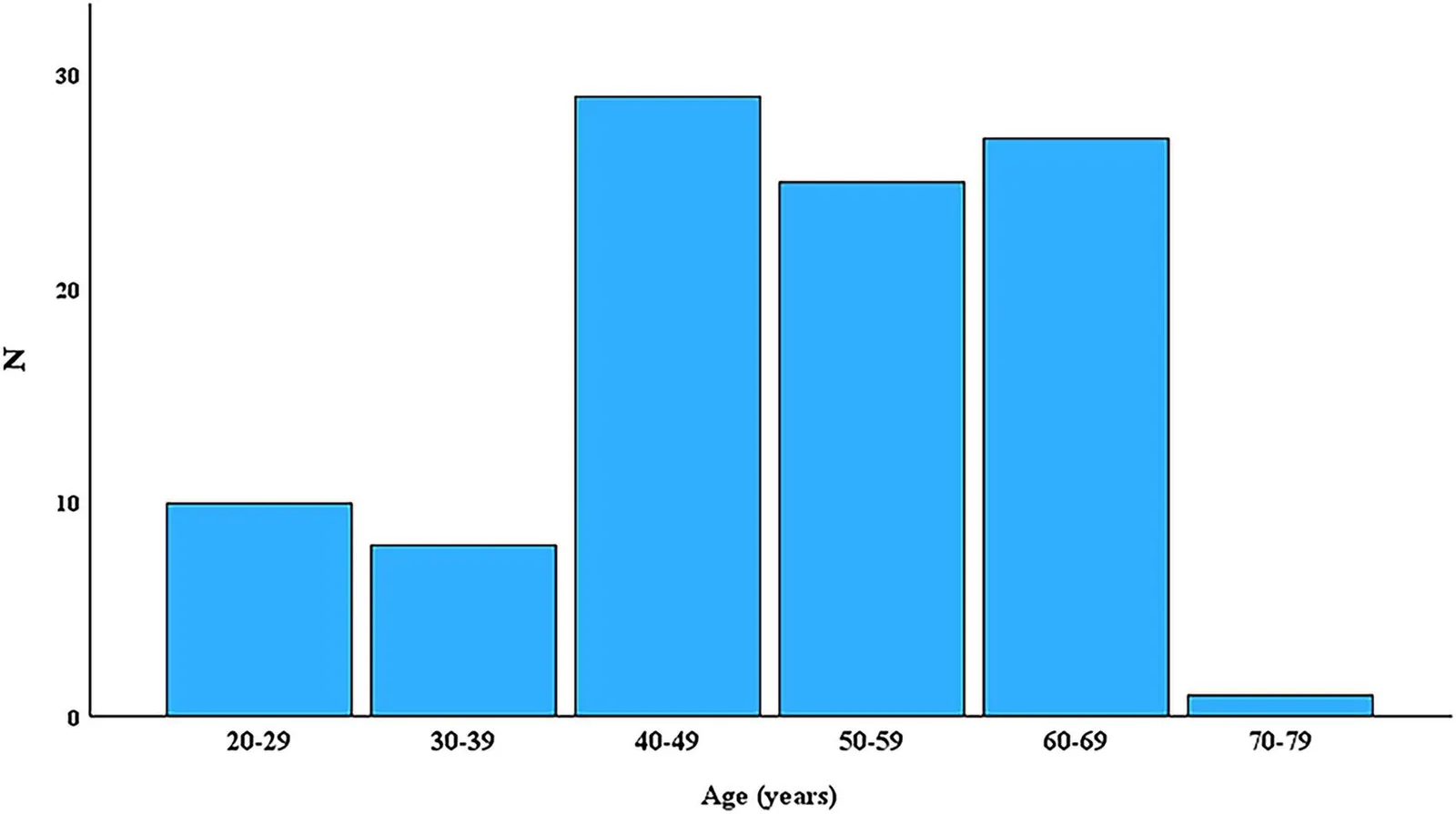
The age distribution diagram of participants.

In total, 100 participants (59 females and 41 males) were included in the study, with a mean age of 49.18 ± 12.47 years (range: 22–72). The mean age for males was 49.49 ± 11.20 years, while for females it was 48.97 ± 13.38 years. No significant difference in age was observed between males and females (*t* = 0.205, *p* = 0.838). All 100 volunteers enrolled exhibited normal cognitive function, as indicated by MMSE scores of ≥ 27 points, with an average score of 28.98 ± 1.10. The age distribution of participants is illustrated in Figure 4. The age-decade-specific normative values are provided in Supplementary Table S1.

### Inter-observer reliability analysis

The ICC of the mean susceptibility values for all ROIs was > 0.942. Given the excellent agreement between the measurements of the two researchers, the final analysis results adopted the average of the measurements from the two researchers.

### The relationship between mean susceptibility values and age

**FIGURE 5:**
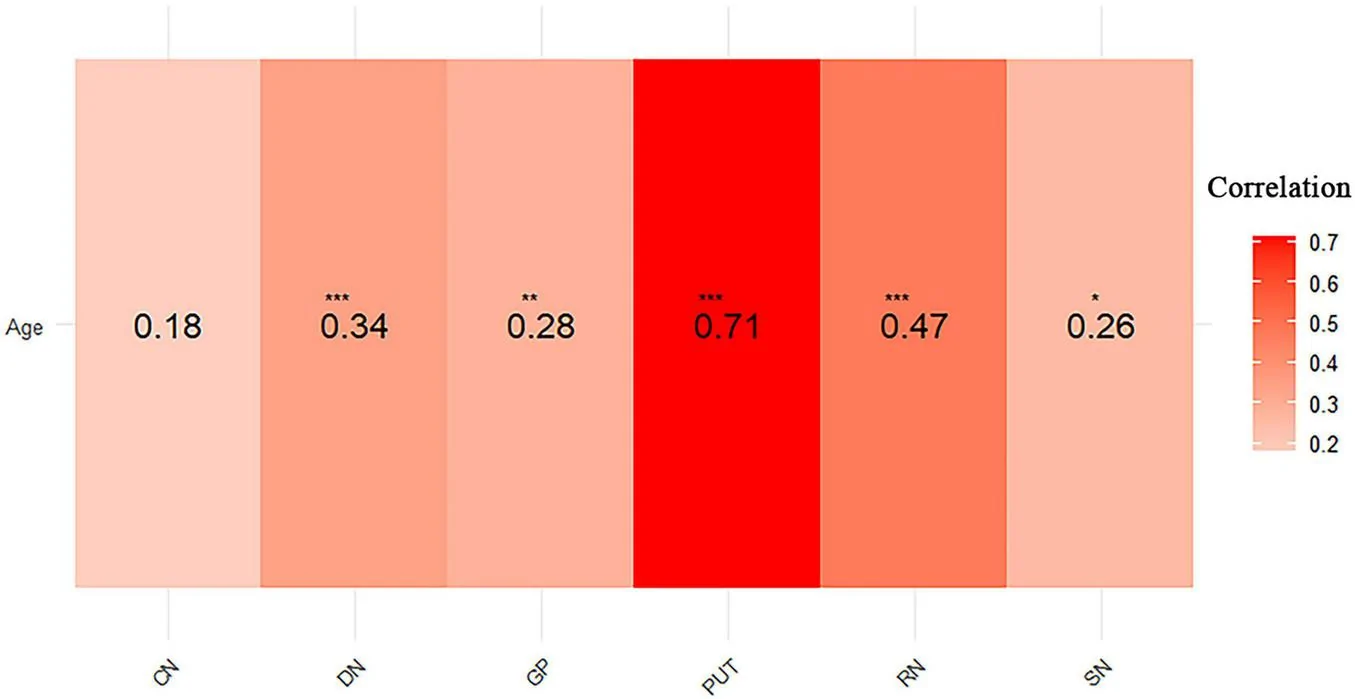
The heatmap of the correlation between age and the susceptibility value of DGM nuclei. Color intensity is coded according to the color bar on the right, with darker colors indicating stronger correlations. *P< 0.05, **P< 0.01, ***P < 0.001.

The mean susceptibility values of the five DGM nuclei except the CN were all positively correlated with age (Table 1 and Figure 5). Among them, the mean susceptibility value of the PUT showed the strongest positive correlation with age (*r* = 0.71, *p* < 0.001), followed by the RN with a moderate positive correlation (*r* = 0.47, *p* < 0.001). The mean susceptibility values of the DN, SN, and GP all showed weak positive correlations with age (DN: *r* = 0.34, *p* < 0.001; GP: *r* = 0.28, *p* < 0.01; SN: *r* = 0.26, *p* = 0.01).

**TABLE 1:** Analysis of the correlation between the mean susceptibility of DGM nuclei and age.

Nuclear	*r*	*p*
RN	0.471	0.000
SN	0.255	0.010
DN	0.342	0.000
CN	0.180	0.074
GP	0.282	0.004
PUT	0.708	0.000

RN, red nucleus; SN, substantia nigra; DN, dentate nuclei; CN, caudate nucleus; GP, globus pallidus; PUT, putamen.

**FIGURE 6:**
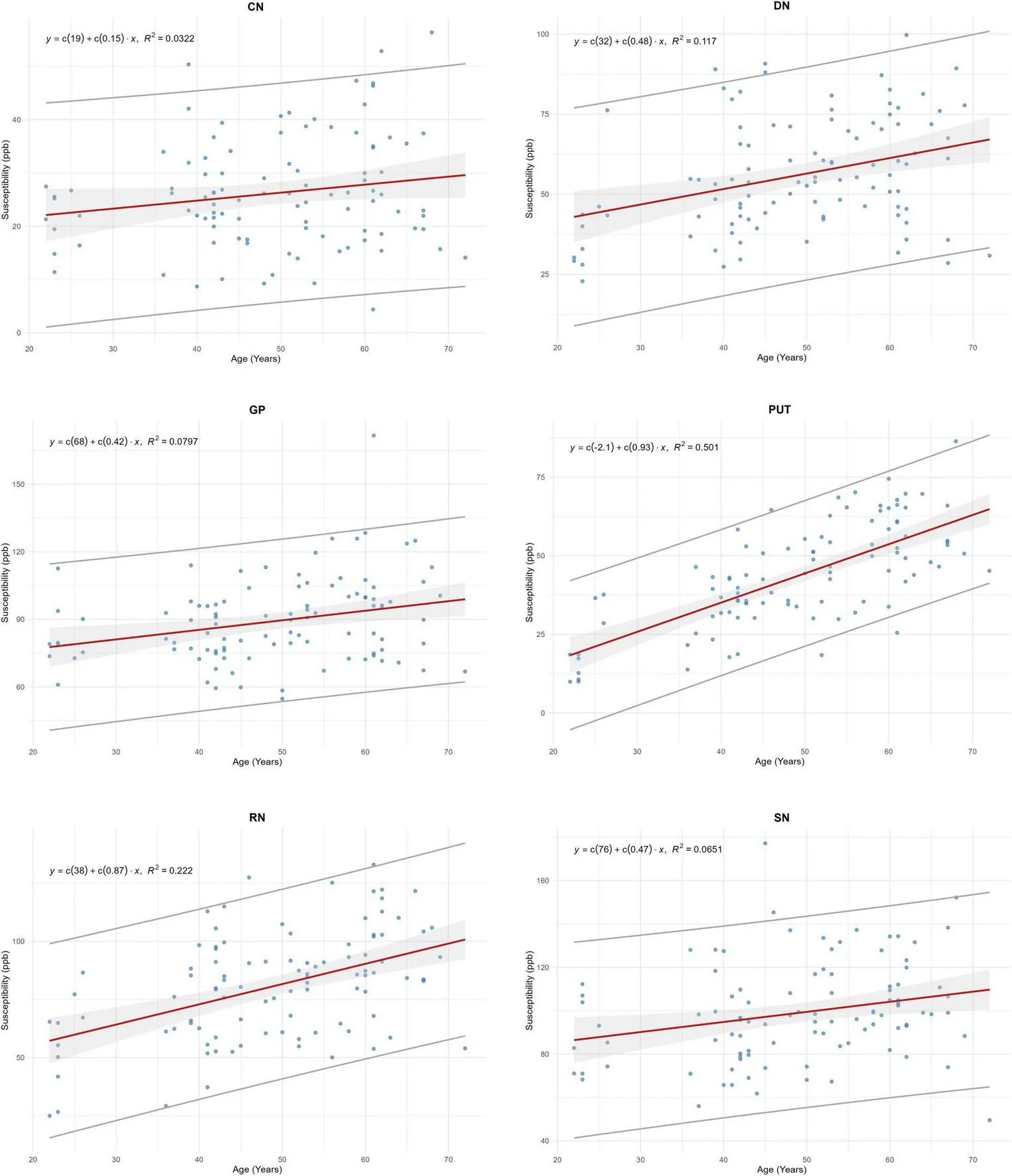
Linear regression fitting of the mean susceptibility value of DGM nuclei with age. Scatter plots and linear regression fits displaying the mean susceptibility values of each DGM nucleus in relation to age are shown. Red line: Linear regression fit of the study data; Gray area: 95% confidence interval; Outermost gray line area: 95% prediction interval. ppb: Parts per billion, 10^–9^.

We performed both linear and quadratic regression analyses to examine the associations between the mean susceptibility values of the six DGM nuclei and age (Supplementary Table S2). Significant linear correlations with age were observed for five of the DGM nuclei, excluding the CN. No significant quadratic relationships with age were identified for any of the DGM nuclei (*p* > 0.05). In addition, except for the DN, the linear model showed lower AIC values than the quadratic model in all other nuclei, and the quadratic model only yielded a minor increase in R^2^. Accordingly, the linear model was used in subsequent analyses (Figure 6).

Analysis of the slopes showed that the rates of increase in susceptibility values of the DGM nuclei with age varied. The mean susceptibility value of the PUT showed the fastest rate of change with age, at 0.93 ppb/year, followed by the RN, DN, SN, and GP, with rates of change of 0.87, 0.48, 0.47, and 0.42 ppb/year, respectively (Table 2).

**TABLE 2:** Linear regression fitting of the mean susceptibility value of DGM nuclei with age.

Nuclear	Slope	Intercept	*R* ^2^	*F*	*p*
DN	0.483	32	0.117	12.959	0.000
RN	0.871	38	0.222	27.936	0.000
SN	0.465	76	0.065	6.821	0.010
CN	0.150	19	0.032	3.265	0.074
GP	0.424	68	0.080	8.482	0.004
PUT	0.930	−2	0.501	98.58	0.000

### Correlations of susceptibility values among multiple nuclei

**FIGURE 7:**
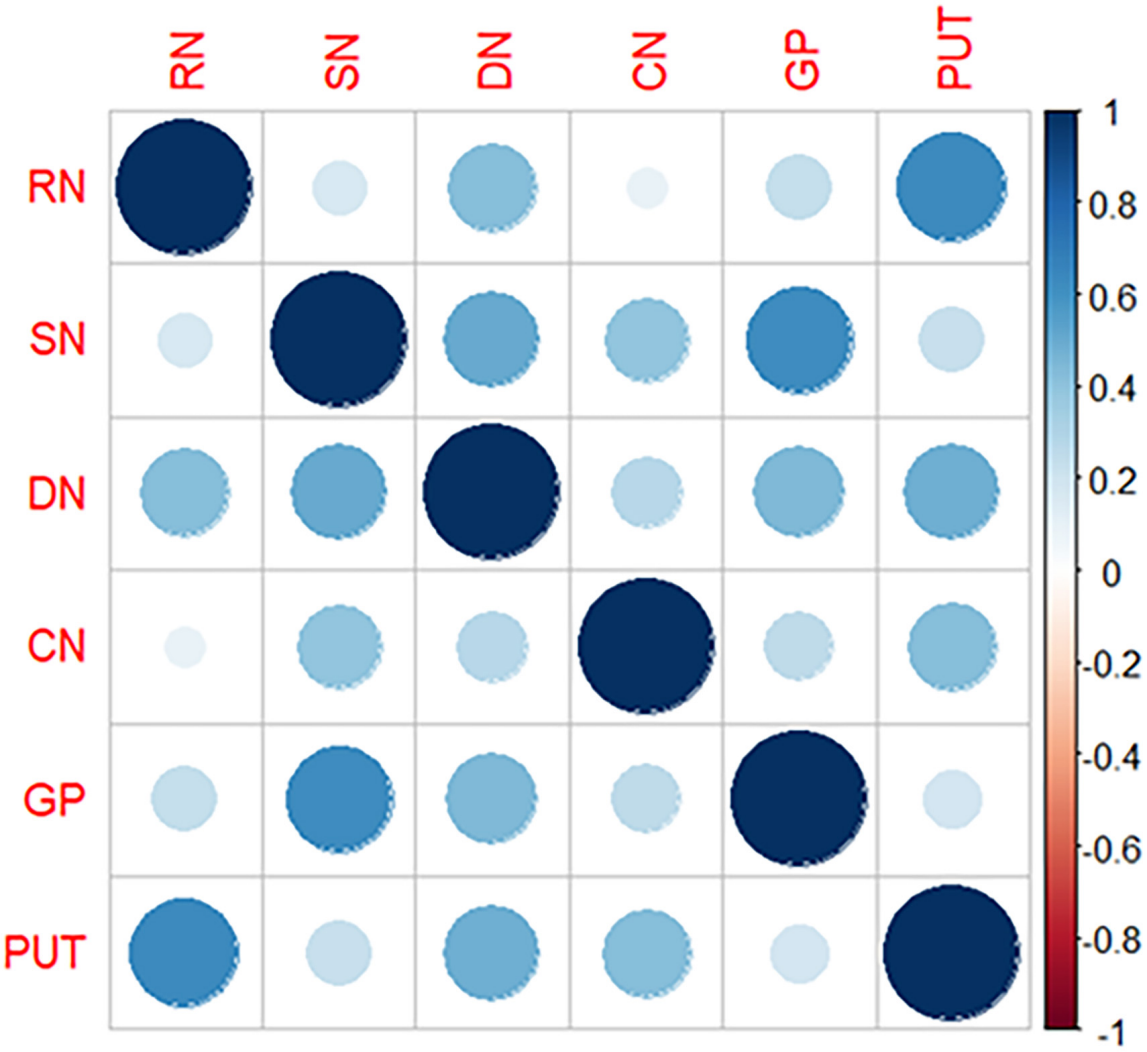
Heatmap of the correlation of the mean susceptibility values among multiple nuclei. Color intensity is coded according to the color bar on the right, with blue indicating positive correlation, red indicating negative correlation, and darker colors representing stronger correlations.

As illustrated in Figure 7, the correlation heat map illustrates the average susceptibility among the DGM nuclei. Table 3 presents the zero-order correlation results prior to age control. Because multiple comparisons were performed, the Bonferroni correction was adopted, establishing a revised significance level of *p* = 0.0033. A strong positive correlation was observed between the average susceptibility values of the RN and PUT (*r* = 0.64, *p* < 0.001), as well as between the SN and GP (*r* = 0.62, *p* < 0.001). Moderate positive correlations were identified between RN and DN (*r* = 0.43, *p* < 0.001), SN and DN (*r* = 0.51, *p* < 0.001), SN and CN (*r* = 0.40, *p* < 0.001), DN and GP (*r* = 0.45, *p* < 0.001), DN and PUT (*r* = 0.49, *p* < 0.001), and CN and PUT (*r* = 0.42, *p* < 0.001). No significant correlations were found between RN and SN, CN or GP; SN and PUT; DN and CN; GP and CN; and GP and PUT (*p* > 0.0033). The remaining nuclear combinations exhibited weak susceptibility correlations.

**TABLE 3:** Correlation matrix of mean susceptibility values among multiple nuclei (zero-order correlation).

Nuclear	RN	SN	DN	CN	GP	PUT
RN	1.000	0.164	0.430**	0.096	0.238	0.636**
SN	0.164	1.000	0.507**	0.395**	0.621**	0.227
DN	0.430**	0.507**	1.000	0.273	0.450**	0.485**
CN	0.096	0.395**	0.273	1.000	0.255	0.421**
GP	0.238	0.621**	0.450**	0.255	1.000	0.186
PUT	0.636**	0.227	0.485**	0.421**	0.186	1.000

**P < 0.001.

Table 4 shows the results of partial correlation analysis with age controlled: The correlations between RN and PUT, and between SN and GP were significantly weakened, shifting from strong to moderate positive correlations (RN vs. PUT: *r* = 0.49, *p* < 0.001; SN vs. GP: *r* = 0.59, *p* < 0.001).The correlation between SN and DN slightly decreased but remained moderate (SN vs. DN: *r* = 0.46, *p* < 0.001). Correlations of RN vs. DN, SN vs. CN, DN vs. GP, and DN vs. PUT weakened from moderate to weak positive (RN vs. DN: *r* = 0.32, *p* < 0.0033; SN vs. CN: *r* = 0.37, *p* < 0.001; DN vs. GP: *r* = 0.39, *p* < 0.001; DN vs. PUT: *r* = 0.37, *p* < 0.001). Notably, the correlation between CN and PUT strengthened while maintaining moderate significance (CN vs. PUT: *r* = 0.42, *p* < 0.001). The correlation between GP and PUT reversed from positive to negative (*r* = −0.021) but remained non-significant (*p* > 0.0033).

**TABLE 4:** Correlation matrix of mean susceptibility values among multiple nuclei after controlling for age.

Nuclear	RN	SN	DN	CN	GP	PUT
RN	1.000	0.052	0.324*	0.013	0.124	0.485**
SN	0.052	1.000	0.462**	0.367**	0.591**	0.068
DN	0.324*	0.462**	1.000	0.229	0.392**	0.366**
CN	0.013	0.367**	0.229	1.000	0.217	0.423**
GP	0.124	0.591**	0.392**	0.217	1.000	−0.021
PUT	0.485**	0.068	0.366**	0.423**	−0.021	1.000

*P < 0.0033,

**P < 0.001.

### Sensitivity analysis: adjustment for sex

Given that previous studies have reported sex differences in brain iron deposition (Li et al., 2023; Peterson et al., 2019), we included sex as a covariate in the model and re-ran linear regression analyses for age and mean susceptibility values, along with partial correlation analyses among nuclei (Supplementary Tables S3, S4).

After adjustment, the significant positive correlations between age and the mean susceptibility values remained stable in the RN (*B* = 0.001, *p* < 0.001), SN (*B* = 0.000, *P* = 0.008), DN (*B* = 0.000, *p* < 0.001), GP (*B* = 0.000, *p* = 0.003) and PUT (*B* = 0.001, *p* < 0.001). The marginal association in the CN (*B* = 0.000, *p* = 0.063) was also unchanged. Meanwhile, after controlling for sex, the correlations between RN and PUT (*r* = 0.49, *p* < 0.001), and between CN and PUT (*r* = 0.43, *p* < 0.001), increased slightly but remained moderately positive. The correlations between SN and DN (*r* = 0.45, *p* < 0.001), and between SN and GP (*r* = 0.57, *p* < 0.001), decreased slightly but also remained moderately positive. Although significant sex effects existed in the SN, CN and GP, sex adjustment did not affect the primary age-related iron deposition associations, nor did it alter internuclear correlations of iron deposition.

## Discussion

This study assessed the relationship between the mean susceptibility values of six DGM nuclei and age in healthy subjects aged 22–72 years, and constructed corresponding age-dependent trajectories. Partial correlation analysis revealed significant inter-correlations among the mean susceptibility values of multiple DGM nuclear groups.

### Iron deposition in DGM nuclei with age

This study found that the mean susceptibility values of five DGM nuclei, with the exception of the CN, exhibited a positive correlation with age. This suggests that iron deposition in DGM nuclei increases as individuals age. This finding aligns with a previous landmark histological study (Hallgren and Sourander, 1958). The correlation strength between average susceptibility values and age differed among various DGM nuclei. The present study found that the PUT showed the strongest positive correlation with age, which is consistent with multiple previous reports (Madden and Merenstein, 2023; Zhou et al., 2020). Followed by the RN with moderate correlation, and the DN, SN, and GP with weak correlations. The relationship between their average susceptibility values and age was not completely aligned with earlier studies.

An early study involving 174 normal subjects aged 20–69 found moderate linear correlations between susceptibility values of the PUT and RN and age (*r* > 0.5, *p* < 0.05), while age dependency was limited in the GP and SN (*r* < 0.25) (Liu et al., 2016), which is generally consistent with the results of this study. However, it found a strong correlation between the CN and age, which is inconsistent with this study. Although a weak correlation between CN and age was also observed in this study, this correlation was not significant (*p* > 0.05). However, a recent study by Howard et al. found that magnetic susceptibility values in the RN and PUT were significantly correlated with age, whereas those in other DGM nuclei such as the SN, GP, CN, and DN showed no significant correlation with age (Howard et al., 2022). The reason for this discrepancy may be due to individual differences. In addition, early studies used susceptibility maps generated from phase images of SWI for correlation studies. SWI often cannot accurately quantify susceptibility values due to blooming artifacts, and its accuracy and sensitivity are inferior to QSM (Meng et al., 2023). Therefore, the above differences may also be caused by different imaging modalities.

Another multicenter investigation that combined data from three different locations discovered a significant positive correlation between mean susceptibility in the CN, PUT, RN, SN, and DN with age (*p* < 0.001). In contrast, the mean susceptibility of the GP exhibited a negative correlation with age; however, this finding was not statistically significant (*p* = 0.49) (Li Y. et al., 2021). Additionally, numerous prior studies have also failed to identify a significant relationship between GP and age (Acosta-Cabronero et al., 2016; Li et al., 2015). However, this study, along with several others (Keuken et al., 2017; Li et al., 2014), found a significant correlation between GP and age, although the strength of correlation varied slightly (*p* > 0.05). A recent longitudinal study with an interval of approximately 8 years also observed a significant age-related increase in GP susceptibility values (Li J. et al., 2021).

Meanwhile, this study observed that the rates of increase in mean susceptibility values of different DGM nuclei with age varied, reflecting heterogeneous iron accumulation within these nuclei. This confirms the findings of an earlier autopsy investigation (Hallgren and Sourander, 1958), which revealed that the distribution of iron within the brain is not uniform, showing the greatest concentration in the basal ganglia and the least within the cortex. Additionally, it was noted that the iron levels in different brain structures rise at varying rates as one ages.

The PUT plays a crucial role in regulating movement and various types of learning (Bernácer et al., 2007; Lim et al., 2019). Currently, numerous studies have demonstrated that iron deposition in the PUT of patients with neurodegenerative diseases is significantly higher than that in age-matched healthy controls. For example, iron deposition is one of the two most prominent neuropathological features of MSA, and it is particularly obvious in the PUT (Lambrecht et al., 2020). In addition, studies have found that the peak iron content in the PUT is associated with the highest density of glial cytoplasmic inclusions and α-synuclein aggregation in the brains of patients with MSA (Jellinger, 2014). This suggests that regions with higher iron content are associated with the primary sites of potential pathology in MSA. Therefore, when investigating iron deposition in DGM nuclei (especially the PUT), the confounding factor of age should be strictly controlled.

Furthermore, the present study found no significant correlation between CN susceptibility and age (*p* > 0.05), and the linear regression model for CN also failed to reach significance (*p* > 0.05). Thus, iron deposition in the CN appears to barely increase with advancing age in our cohort—a finding consistent with the cross-sectional study by Li et al., which included 132 healthy adults aged 40–83 years (Li et al., 2015). Additionally, a 2-year longitudinal study of young and middle-aged healthy controls, conducted in the context of neurodegenerative disease, reported significant iron accumulation in the PUT and GP, but no such increase in the CN (Walsh et al., 2014). However, previous studies reported mean annual susceptibility changes in the CN ranging from 0.28 to 0.82 ppb/year (Acosta-Cabronero et al., 2016; Li Y. et al., 2021; Liu et al., 2016). A recent large-scale study of 498 healthy individuals aged 5–90 years may explain this discrepancy (Treit et al., 2021). That study found that CN mean susceptibility reached 90% of its total change by age 36 and plateaued thereafter. The healthy volunteers in the present study had a mean age of 49.18 ± 12.47 years, with an age distribution concentrated between 40 and 70 years. As noted above, CN susceptibility plateaus in the late 30s, which readily explains why our study observed only minimal age-related changes in CN iron deposition.

### Correlation of iron deposits among nuclear groups

This study analyzed the correlations of mean susceptibility values among DGM nuclei, and found significant correlations between multiple nuclear groups such as RN and PUT, SN and DN, SN and GP, etc. These results are mostly consistent with a previous study (Persson et al., 2015), though some discrepancies exist. For example, this study revealed a significant positive correlation in mean susceptibility values between DN and PUT, which was not observed in the previous study. Conversely, that study found significant positive correlations between RN and SN, RN and CN, while no significant correlations were detected in this study. The observed differences are speculated to result from variations in participants’ age, gender, and demographic distribution. Additionally, before controlling for age, both this study and the previous research observed significant positive correlations in mean susceptibility values between RN and GP, SN and PUT. However, the present study found that these correlations became nonsignificant following either Bonferroni correction or adjustment for age, indicating that iron deposition in these nuclei is independently influenced by age rather than arising from a synergistic effect.

A notable finding is that this study revealed persistent significant positive correlations in mean susceptibility values between SN and GP, SN and CN, as well as PUT and CN after age and sex control, which readily evokes associations with the nigrostriatal dopaminergic pathway involving these nuclei and the role of iron in this pathway.

The nigrostriatal dopaminergic pathway is a neural circuit projecting from dopaminergic neurons in the SN to the dorsal striatum (CN and PUT), playing a critical role in motor function control and learning ability (Hikosaka et al., 2002). PD is the most common disorder involving dopamine signaling dysfunction, which is pathologically characterized by the progressive loss of dopamine-producing neurons in the substantia nigra pars compacta (SNc) and the formation of Lewy bodies abundant in α-synuclein (Kalia and Lang, 2015). The disease manifests as a group of motor symptoms termed parkinsonism, including bradykinesia, resting tremor, rigidity, and postural instability, which are typically attributed to the loss of dopamine in the striatum (Fearnley and Lees, 1991). Dopamine metabolism involves multiple pathways, not all of which have been fully elucidated; these pathways generate neurotoxic substances, several of which are iron-dependent. Iron overload can induce the production of reactive oxygen species (ROS) via the Fenton reaction, leading to oxidative stress and cell death (Salvador, 2010). In addition, iron overload causes the oxidation of dopamine that is not taken up by synaptic vesicles in the cytoplasm into DA-o-quinone, aminochromes, and 5,6-indolequinone. Such quinone metabolites can induce mitochondrial dysfunction, α-synuclein aggregation, and oxidative stress, and play an important role in the degenerative process (Aguirre et al., 2012; Hauser and Hastings, 2013; Muñoz et al., 2012).

Increased iron content in the substantia nigra pars compacta (SNc) of patients with PD has been confirmed by numerous studies (Alushaj et al., 2024; Guo et al., 2024). However, no prior study has examined the correlations of iron deposition among the DGM nuclei that are closely related to the nigrostriatal dopaminergic pathway in PD patients. A multimodal MRI study reported significantly reduced functional connectivity between the bilateral striatum in patients with PD, and this reduced functional connectivity was negatively correlated with iron content in the lower part of the substantia nigra (Guan et al., 2019). It remains unclear whether abnormal iron deposition in the substantia nigra affects the correlations of iron deposition among several DGM nuclei involved in the nigrostriatal dopaminergic pathway in patients with PD. The present study provides normal control data for further investigations in PD patients in the future.

Significant correlations were observed among the mean magnetic susceptibility values of multiple DGM nuclei, which may indicate that strongly interconnected nuclei share similar iron deposition patterns or common pathological mechanisms. This observation offers new perspectives for investigating brain iron deposition. For example, whereas previous studies have largely focused on individual nuclei, the correlation analysis of iron deposition presented here suggests that strongly associated nuclei could be examined as an integrated iron metabolic unit in future research.

Previous studies conducted on cell lines have demonstrated that the mechanistic target of rapamycin (mTOR) plays a crucial role in regulating cellular iron homeostasis (Conjard-Duplany et al., 2025), and aberrant activation of the mTOR signaling pathway can disrupt the dynamic equilibrium of cellular metabolism (Zhao et al., 2024). Moreover, mTOR-dependent signaling pathways are **c**losely associated with neurodegenerative diseases such as Alzheimer’s disease and Parkinson’s disease (Fan and Xu, 2026; Malagelada et al., 2006). We hypothesize that the highly correlated iron deposition among DGM nuclei in healthy individuals may be coordinately mediated by the mTOR signaling pathway, rather than being independently regulated. Furthermore, the highly correlated pattern of iron deposition across DGM nuclei in neurodegenerative disorders may share dysregulation of the mTOR pathway as a common upstream driver.

### Limitations

The age distribution of the sample was concentrated in the 40–70 year range, accounting for 79 of 100 participants. Only one subject was over 70 years old, and the number of younger participants was relatively small. This uneven distribution may have biased the regression estimates of age-related susceptibility changes, particularly at the lower and upper ends of the age spectrum. The scarcity of elderly participants limited our ability to characterize potential plateauing or reductions in iron deposition in older adults, while the small number of younger participants hindered the detection of early age-related changes. Consequently, our findings are most robust for middle-aged adults (40–70 years), and generalization to younger or older populations should be undertaken with caution. Future studies should employ larger, more balanced samples that cover a broader age range to elucidate lifespan changes in iron deposition within DGM nuclei.

## Conclusion

In summary, this QSM study provides an age-dependent trajectory of iron deposition in DGM nuclei of healthy populations during aging, suggesting that strongly correlated nuclei may share similar iron deposition patterns or pathological mechanisms. These findings provide a reference and offer new perspectives for investigating iron deposition in neurological disorders.

## Data Availability

The original contributions presented in the study are included in the article/Supplementary material, further inquiries can be directed to the corresponding authors.

## Footnotes

1
https://people.eecs.berkeley.edu/chunlei.liu/software.html
