# Supplementary material for: Using QSM to investigate the iron deposition patterns in deep gray matter nuclei during the aging process in healthy populations
Source: Front Aging Neurosci. 2026 Aug 18;18:1839435. doi: 10.3389/fnagi.2026.1839435 (PMC13529694; doi:10.3389/fnagi.2026.1839435)
Supplement: Supplementary file 1 [file Data_Sheet_1.pdf]

Table S1

Mean susceptibility values (ppm) of deep gray matter nuclei stratified by age decade.

Age (years)	N	RN	SN	DN	CN	GP	PUT
20-29	10	0.056±0.220	0.087±0.016	0.039±0.015	0.021±0.005	0.0817±0.014	0.020±0.011
30-39	8	0.067±0.018	0.098±0.026	0.052±0.017	0.031±0.012	0.089±0.013	0.030±0.012
40-49	29	0.080±0.022	0.095±0.026	0.056±0.018	0.023±0.008	0.083±0.014	0.039±0.010
50-59	25	0.081±0.018	0.101±0.020	0.059±0.013	0.028±0.010	0.093±0.019	0.050±0.014
60-69	27	0.096±0.020	0.107±0.019	0.061±0.019	0.029±0.012	0.097±0.023	0.056±0.013

Table S2

Comparisons of Linear and Quadratic Regression Models for DGM Nuclei and Age

DGM Nucleus	Linear			Quadratic		
	R^2	*p*	AIC	R^2	*p*	AIC
RN	0.222	0.000	-490.164	0.234	0.222	-489.710
SN	0.065	0.010	-474.489	0.069	0.527	-472.904
DN	0.117	0.000	-531.133	0.000	0.062	-532.751
CN	0.032	0.074	-627.343	0.034	0.642	-625.566
GP	0.080	0.004	-514.592	0.081	0.682	-513.016
PUT	0.501	0.000	-603.209	0.505	0.385	-601.991

AIC, Akaike information criterion.

Table S3

Linear regression fitting of the mean susceptibility value of DGM nuclei with age adjusted for sex

	Age		Sex	
	B	*p*	B	*p*
RN	0.001	0.000	0.006	0.152
SN	0.000	0.008	-0.011	0.016
DN	0.000	0.000	-0.004	0.203
CN	0.000	0.063	-0.004	0.043
GP	0.000	0.003	-0.009	0.012
PUT	0.001	0.000	-0.019	0.789

B, Unstandardized Coefficients B

Table S4

Correlation matrix of mean susceptibility values among multiple nuclei after controlling for age and sex

	RN	SN	DN	CN	GP	PUT
RN	1.000	0.090	0.350**	0.044	0.167	0.494**
SN	0.090	1.000	0.448**	0.334**	0.565**	0.063
DN	0.350**	0.448**	1.000	0.209	0.374**	0.366**
CN	0.044	0.334**	0.209	1.000	0.175	0.427**
GP	0.167	0.565**	0.374**	0.175	1.000	-0.028
PUT	0.494**	0.063	0.366**	0.427**	-0.028	1.000

*indicates $P < 0.0033$, **indicates $P < 0.001$.
